# Targeted Single-Phage Isolation Reveals Phage-Dependent Heterogeneous Infection Dynamics

**DOI:** 10.1128/spectrum.05149-22

**Published:** 2023-04-17

**Authors:** Magdalena Unterer, Mohammadali Khan Mirzaei, Li Deng

**Affiliations:** a Institute of Virology, Helmholtz Centre Munich, German Research Centre for Environmental Health, Neuherberg, Germany; b Chair for Prevention of Microbial Infectious Diseases, School of Life Sciences, Technical University of Munich, Freising, Germany; University of Exeter

**Keywords:** bacteriophages, flow cytometry, single-cell analysis

## Abstract

Due to rising antibiotic resistance, there is an urgent need for different treatment options for multidrug-resistant infections. One alternative under investigation is phage therapy, which uses phages to treat bacterial infections. Although phages are highly abundant in the environment, not all phages are suitable for phage therapy, and finding efficient phages that lack undesirable traits such as bacterial virulence factors is challenging. Here, we developed a targeted single-phage isolation method to detect and isolate phages of interest and to characterize their kinetics in a high-throughput manner. This assay has also revealed cell-to-cell variations at a single-cell level among cells infected with the same phage species, as well as among cells infected with different phage species.

**IMPORTANCE** The spread of multidrug-resistant bacteria is a global human health threat, and without immediate action we are fast approaching a postantibiotic era. One possible alternative to antibiotics is the use of phages, that is, bacterial viruses. However, the isolation of phages that effectively kill their target bacteria has proven challenging. In addition, isolated phages must go through significant characterization before their efficacy is measured. The method developed in this work can isolate single phage particles on the basis of their similarity to previously characterized phages while excluding those with known undesirable traits, such as bacterial toxins, as well as characterizing their kinetics. Using this method, we revealed significant cell-to-cell variations in phage kinetics at a single-cell level among highly virulent phages. These results shed some light on unknown phage-bacterium interactions at the single-cell level.

## OBSERVATION

The idea of using bacteriophages or phages to treat bacterial infections is experiencing a rebirth due to the increased development of multidrug-resistant bacteria (1, 2). Phages can be found in the environment in large quantities; there are an estimated 10^31^ phages on Earth, but not all phages are suitable for phage therapy (2, 3). Phages are mainly isolated using culture-based methods, which have some limitations and can favor phages with specific infection properties, such as a short latency period or a particular optimum temperature or pH for replication (4). Culture-independent methods such as viral tagging may address these issues (5).

**FIG 1:**
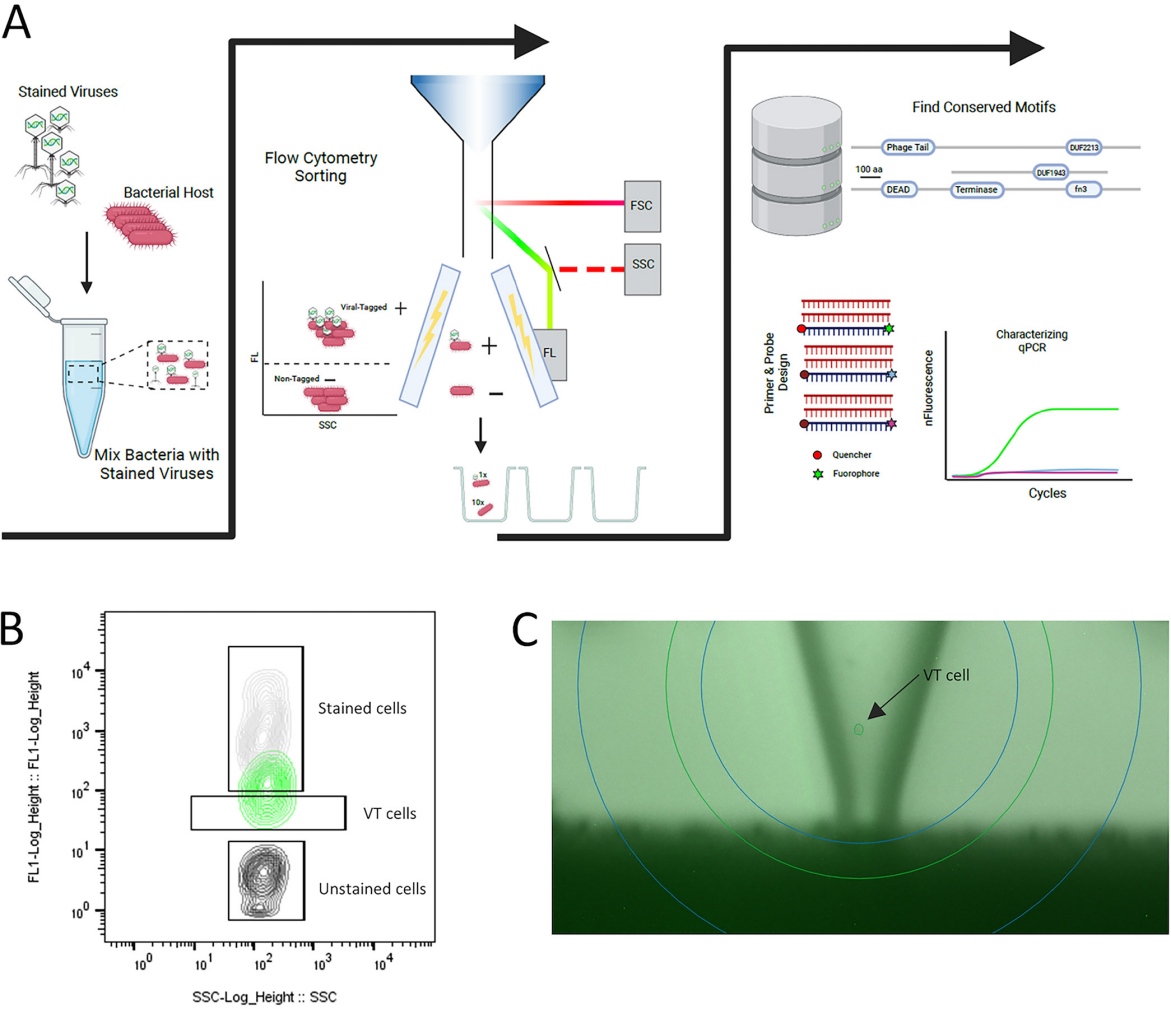
Targeted single-phage isolation method. (A) Overview of experimental setup. Stained VLPs are mixed with target bacteria and are sorted in bacteria-containing wells based on their thresholds from the forward scatter (FSC), the side scatter (SSC), and fluorescence (FL). Growth kinetics are monitored, and phage properties are subsequently identified via multiplex qPCR. (B) Viral tagging plot of E. coli infected by T4. The merged plot was generated by a flow cytometer. Unstained cells are shown in black, stained cells in gray, and virally tagged (VT) cells in green. The *y* axis shows the fluorescence level and the *x* axis the side scatter. (C) Viral tagging on the microfluidics device. The camera shows 1 virally tagged cell (arrow) (E. coli with T4), which was selected by the microfluidics device to be sorted.

Viral tagging uses nucleic acid dyes to stain virus-like particles (VLPs), differentiating infected from noninfected cells using fluorescence intensity. Virally tagged cells are separated via fluorescence-activated cell sorting (FACS), and phage-host pairs are identified by sequencing (5–7). In this study, we developed a new approach for (i) the targeted isolation of phages (Fig. 1A and B) and (ii) the assessment of their efficiency at a single-cell level using viral tagging (see Fig. S3A, S4A and S5A in the supplemental material). The latter was added to assess population heterogeneity, which is overlooked by the classic characterization methods, and to identify subpopulations that may survive phage infection.

The FACS process, e.g., pressurized sheath fluid, can introduce external stressors. In order to minimize this, we adapted viral tagging to a microfluidic device that uses gravity to sort its droplets, each of which contains a single bacterium-phage pair (Fig. 1C; also see Fig. S1A to E). We selected T1, T4, and T7 Escherichia coli phages to evaluate the feasibility of the method developed. The three phages infect the same host, represent the major tailed phage families, i.e., *Siphoviridae*, *Myoviridae*, and *Podoviridae*, and are highly efficient against their hosts and variably host specific (8–10). In addition, none of these phages carries virulence genes, which makes them plausible candidates for phage therapy (11).

Single virally tagged phage-host pairs (Escherichia coli 11303 with T1, T4, or T7) were sorted in a 96-well plate, in which each well contained 10 sorted E. coli cells, and growth kinetics were monitored overnight using optical density (OD) measurements. We next used multiplex quantitative PCR (qPCR) to identify the sorted pairs based on the following selection criteria: (i) they should include conserved structural proteins of T1, T4, and T7, and (ii) they should lack the toxin genes and virulence factors (see Fig. S2A to E) that are carried by some coliphages (12). These criteria were chosen to establish the method and can be adapted to different bacterial taxa to avoid phages with virulence genes unsuitable for therapy. The chosen virulence factors were based on the currently most common virulence factors carried by coliphages (12).

Despite the use of similar settings and an isogenic bacterial population picked from a single colony, cells showed different behaviors; 78.8% of the wells showed a productive infection when T1 was used (see Fig. S3A), 42.3% of the wells were positive with T4, and 5.9% were positive with T7 (see Fig. S4A and Fig. S5A). For each phage, 30 wells were analyzed by multiplex qPCR. All targets were amplified without any cross-amplification. In addition, no signal loss was observed if multiple targets were amplified in the same well (see Fig. S3 to Fig. S5B and C). We next tested the specificity of our method using wastewater spiked with three different concentrations of the three coliphages, i.e., 1,000× higher than the concentration of wastewater VLPs, equal to the concentration of wastewater VLPs, or 1,000× lower than the concentration of wastewater VLPs. However, we detected the spiked phages only at the highest concentration. To check whether this is a limitation of single-cell sorting, we repeated the experiment using a flow cytometer and sorted 1 million cells (see Fig. S6A and Fig. S7). Here, we could identify the phage in wastewater regardless of its concentration (see Fig. S6B). Similar results were seen when a mixture of all three phages (1:1:1 ratio) was sorted. T1 was detected by both single-cell and population-level sorting, whereas T4 and T7 were identified only when cells were sorted in bulk (see Fig. S6C and D). To explore how the three phages interacted with their common host, we then closely analyzed their single-cell growth kinetics.

**FIG 2:**
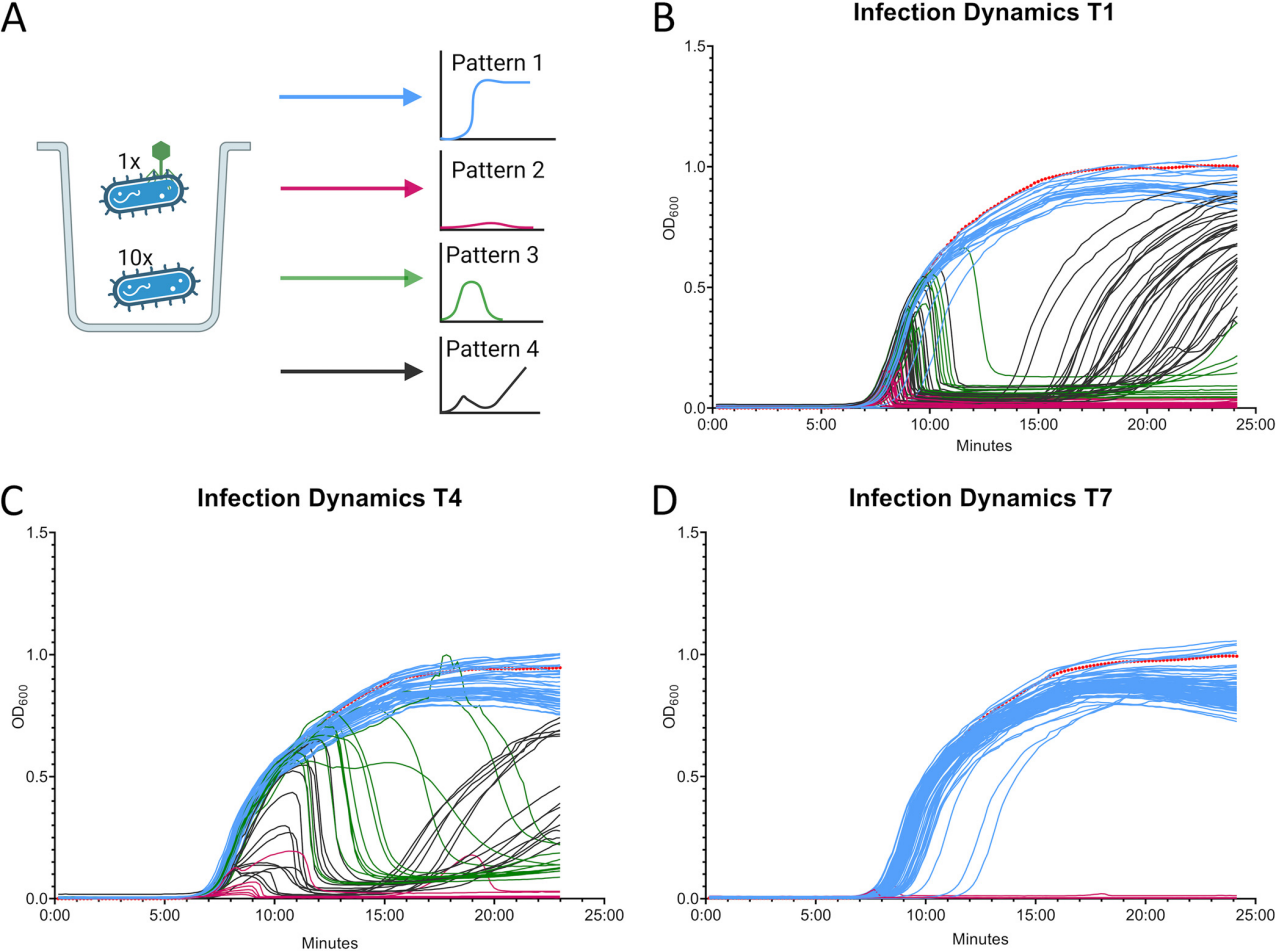
Cell-to-cell variations observed in phage-bacterium interactions. (A) Schematic overview of possible infection dynamics from single virally tagged cells (1 virally tagged cell plus 10 bacterial cells). Samples with no lysis are presented in blue (pattern 1). Pattern 2 includes samples with early lysis, which are shown in pink. Samples with early bacterial growth and late lysis are shown in green and referred to as pattern 3. Pattern 4 includes samples with early lysis but bacterial regrowth, which are shown in dark gray; all 17 colonies picked with this condition were resistant to the phage with which they were initially infected, showing the growth of resistant phenotypes. Wells were prefilled with 10 bacterial cells, and 1 virally tagged cell was added to them. Plates were incubated overnight, and infection dynamics were monitored via OD_600_ measurements. (B) T1-infected cells. (C) T4-infected cells. (D) T7-infected cells. In panels B, C, and D, the different infection patterns are color-coded as in panel A. In each analysis, a bacterium-only control was also monitored and is shown in red in the graphs.

We compared single-cell growth curves and calculated the area under the curve (AUC), and we found significant cell-to-cell variations, with four distinct patterns, i.e., (i) no lysis (Fig. 2A to D, blue), (ii) total lysis (Fig. 2A to D, pink), (iii) late lysis (Fig. 2A to D, green), and (iv) early lysis with late bacterial recovery (Fig. 2A to D, dark gray). The majority of T1-infected cells had low or median AUC values (see Fig. S8A), indicating total lysis. This aligns with literature studies that reported high killing efficiency for T1 at the population level (8, 13). T4 showed higher AUC values (see Fig. S8B), suggesting a low lysing efficiency for single cells, which is in contrast to earlier studies at the population level (14, 15). The same is true for T7 (15), because we observed only no lysis or total lysis at the single-cell level (see Fig. S8C). When the lysis patterns were compared within one phage sample, T1- and T7-infected cells showed little to no sample diversity, whereas T4 had high cell-to-cell variation. To test whether our experimental conditions were influencing the infection properties of the phages, one-step growth curves were analyzed (see Fig. S2F to H). We did not observe significant differences in the latency period and the burst size of the phages under our experimental conditions, compared to standard conditions. These results highlight the complexity of phage-bacterium interactions at the single-cell level, which might partially explain inconclusive results with the therapeutic application of phages (16).

### Conclusion.

The transition of viral tagging from flow cytometry to a microfluidics device opens up new possibilities and allows for true single-cell sorting while limiting the stresses usually induced by flow cytometry. Using this method, we could identify significant variations in phage-bacterium dynamics among coliphages at the single-cell level that were previously unknown. This is specifically important for the success of phage therapy, because cell-cell heterogeneity plays a role in drug susceptibility. The genotypic and phenotypic heterogeneity observed at the single-cell level in bacteria may partially explain our results (17, 18). However, it must be considered that the presence of 10 bacterial cells preloaded in each well before sorting of the single virally tagged cells, which is necessary to allow the phage to replicate, precludes truly identifying phage-bacterium dynamics at the single-phage/single-bacterium level. However, we observed large variations in infection patterns for different phages, suggesting heterogenicity in phage-bacterium interactions. Combining our single-cell isolation method with omics approaches could shed light on the mechanisms underlying the observed cell-cell variations and significantly advance our understanding of phage-bacterium interactions. In addition, due to its high flexibility, this method can be used under different cultivation conditions, including aerobic and anaerobic conditions (19). The technique can also be used for the targeted isolation of not-yet-cultivated phages that have been identified in metagenomic data but have proved to be highly challenging to isolate, such as CrAssphages (20).
