# Supplementary material for: Targeted Single-Phage Isolation Reveals Phage-Dependent Heterogeneous Infection Dynamics
Source: Microbiol Spectr. 2023 Apr 17;11(3):e05149-22. doi: 10.1128/spectrum.05149-22 (PMC10269501; doi:10.1128/spectrum.05149-22)
Supplement: Supplemental file 1 — Supplemental material. Download spectrum.05149-22-s0001.pdf, PDF file, 2.9 MB [file spectrum.05149-22-s0001.pdf]

Supplementary Data:

1 Methods

1.1 Bacteria and Bacteriophages

A single colony of *Escherichia coli* 11303 was cultured in Luria Bertani broth (Carl Roth, X968.1) overnight at 37 °C shaking at 150 rpm for each experiment.

For phage propagation, phage stock was added to bacterial culture in exponential phase and incubated overnight. The enriched culture was centrifuged at 6000 g for 15 minutes before the supernatant was filtered with a 0.22 µm sterile filter (Merck, Millex-GP, SLGP033RS). Phage titer was determined using a double-layer plaque assay. 3 mL LB soft agar (0.6 %) was mixed with 200 µL bacterial overnight culture and 100 µL phage dilution, poured onto an agar plate (1.5 %) and incubated overnight at 37 °C. Plaques were counted and titer was calculated. Phage stocks were stored at 4 °C until further use.

1.2 Viral Tagging and Single Cell /Bulk Sort

Single Cell Viral Tagging:

Phage supernatant was mixed with Syto9 (ThermoFisher, S34854) and incubated in the dark at room temperature for 30 minutes. Stained VLPs were washed three times with an ultrafiltration unit (Vivaspin 20, 100.000 MWCO PES, VS2042) and water (Milli-Q) at 3000 g for 3 minutes. Washed samples were stored on ice. Bacterial overnight culture was washed three times with

0.9 % NaCl solution at 5000 g for 3 minutes. Bacteria and stained virus-like particles (VLPs) were mixed together and incubated at 37 °C for 5 minutes, shaking at 500 rpm.

Single-cell sorts were performed with the single-cell dispenser bf.sight (Cytena). The bf.sight is a micro-fluidics device that generates droplets monitored with a camera to detect cells inside the droplet. If a single cell is inside the droplet and fulfils all pre-set requirements, the droplet will be dispensed. Additionally, to the camera and bright-field mode, the device is equipped with a laser and a fluorescence detector enabling the differentiation between fluorescently stained and unstained particles. Before each experiment, a droplet quality control check was performed according to the manufacturer's instructions. If needed, the droplet was repositioned and aligned to the camera's center to ensure a stable and focused cell sort. The right sample concentration was checked visually and adjusted when necessary with higher or lower dilutions. The optimal starting bacterial cell concentration was found to be 10^5 CFU/mL. However, each sample was assessed to get an estimation of the number of cells in the cartridge by observing b. sight's camera. If necessary, sample concentration was increased by adding more bacteria or diluted by adding water inside the sorting cartridge. The right sample concentration is crucial to achieve an even and stable sort and avoid cartridge blockages which means that droplet formation is hindered and sorting is not possible. Brightfield-Fluorescence (bf) mode was selected for sorting to increase specificity. This mode verifies bright-field settings (size was set between 0.8 and 3, roundness was set between 0.5 and 1) before determining the fluorescence status (FL Intensity between 46-56 and FL Size between 0-10) of the cell. Ten bacterial cells were sorted in 96-wells pre-filled with 200 µl LB and one viral tagged cell was sorted into these wells. The plate was

incubated overnight at 37 °C with continuous shaking in the plate reader (BioTek EPOCH2) to follow OD_{600}-changes over time. To compare individual growth curves, the area under curve was calculated with the assistance of GraphPad Prism Version 8.4.3. (see formula below)(1)

$$\int_a^b f(x) = \sum_{i=1}^{n} \frac{f(x_{i-1}) + f(x_i)}{2} * \Delta x_i$$

$$= \frac{0.5}{2} (f(x_0) + 2f(x_1) + 2f(x_2) + \cdots + 2f(x_{n-1}) + f(x_n))$$

Δ x … time interval (30 min = 0.5)

n … number of total OD_{600} measurements

$f(x_0)$ … first OD_{600} measurement

$f(x_N)$ … last OD_{600} measurement

Bulk Sort Viral Tagging:

For bulk sorting, additional controls were prepared. In short, two 1 mL overnight bacterial aliquots were washed three times with 0.9 % NaCl solution at 5000 g for 3 minutes, before one aliquot was stained for 30 minutes with 1 µL Syto9. After staining, each aliquot was washed again three times. Virus-like particles were prepared as described previously in stained and unstained form. 1 mL of Milli-Q water was mixed with 1 µL Syto9 and processed in the same way as VLPs for a buffer control. Bacteria were incubated once with stained VLPs and once with unstained VLPs at 37 °C, for five minutes shaking at 500rpm. All samples were diluted with H_2O to the appropriate dilution for the flow cytometer MoFlo XPD (Beckman Coulter). Before processing all

samples, the laser was aligned using Quality Control Beads (CytoFLEX Daily QC Fluorospheres, Beckmann Coulter, B53230) and IntelliSort was activated for sorting. To avoid any fluorescence spill overs, samples were processed in the following order: unstained buffer, unstained VLPs, unstained bacteria, unstained bacteria with unstained VLPs, stained buffer, stained VLPs, stained bacteria. Between each sample the sample-line was back flushed and between unstained and stained samples the sample line was cleaned with water for 3 minutes. The sample line was also washed before the viral tagged sample. Each sample was run for 1 minute before data was recorded either for 10 seconds or 10,000 events. Thresholds were set on Side Scatter Threshold 0.06 based on buffer background noise and bacterial size. Events were visualized on bi-exponential plots and a parental gate was drawn in the forward vs side scatter plot to ensure that only bacterial cells appear in the daughter plots. Within daughter plots, stained and unstained bacteria were gated to define minimal fluorescence and auto-fluorescence, respectively. The sort gate was set in between to avoid any overlaps with the stained or unstained cells. 1 million cells were sorted using a low flow rate to guarantee high purity. Sorted cells were stored at 4°C until further use. Data files (.fcs) were processed using FlowJo (10.8.1 CL).

Sample Spiking:

240 mL wastewater was filtered through a 0.22 µm filter and centrifuged for 3 hours at 35.000 g. Pellets were resuspended in 5 mL of PBS and filtered again. To determine VLP concentration, 1 mL of concentrated wastewater was stained with SybrGold (50x SybrGold 1 µL, 30 °C, 30 minutes), diluted with PBS and ran on a Nanoparticle Tracking Analysis (NTA, NanoSight, Malvern Panalytical) device. 10^8 to 10^9 virus-like particles per milliliter were detected and phage virus-like

particles were either added 1000x more, in equal concentrations or 1000x less than VLPs from the wastewater.

1.3 Phage Characterization

Primers and probes used in this study are listed in Supplementary Tables 1 & 2. Primers and probes were designed based on conserved motifs from phage genes after blastx search in NCBI and created using PrimerQuest Tool from Integrated DNA Technologies. Cy5, FAM and HEX were chosen as fluorophores, and BBQ650 and BHQ1 as quenchers, respectively. DINAMelt Server was used to check for primer hairpins and dimers. qPCR was performed on Agilent Mx3000P using the Agilent Brilliant Probe Multiplex MM (Agilent, 600553). Each reaction contained all three primer sets (at 300 nM), three probes (at 100 nM), 1x master mix, reference dye and water with a final reaction volume of 20 µL. Master mix and reference dye were used as described in the manual. 1 µL of sample (1:10 dilution) was added. The qPCR protocol included a 10 minute 95 °C initial activation step followed by 40 cycles of 95 °C for 15 seconds and then 1 minute at 62 °C.

For analysis, normalized data generated by the qPCR machine was used. Each plate had standards for each primer set including a range of concentrations from 10 ng DNA to 0.0001 ng DNA. DNA was extracted using the Norgen Phage DNA Isolation Kit (Norgen Biotek Corp., Cat. #46800) for phage DNA and DNeasy PowerLyzer Microbial Kit (Quiagen, Cat. No. / ID: 12255-50) for bacterial DNA. Lambda phage DNA was used as a negative control with 1 ng and as a no-template control, water was added. Standards and negative controls were performed in

triplicate. DNA standards were generated via serial dilution ranging from 10 ng to 0.1 pg. Sample supernatant was diluted (1:10) with PCR-grade water.

1.4 Phage quantification

Phage quantification was done with a double-layer spot assay. In short, 3 mL LB soft agar (0.6 %) was mixed with 200 µL bacterial overnight culture and poured onto an agar plate (1.5 %). 10 µL of serially diluted phage supernatant was spotted onto the solid soft agar and the plates were incubated overnight at 37 °C.

1.5 Syto9 toxicity

To test Syto9 toxicity, 1 mL phage supernatant was stained with 1 µL Syto9 and incubated for 30 minutes at room temperature. After incubation, phage was washed three times with water (Milli-Q) in an ultrafiltration unit (Vivaspin 20, 100.000 MWCO PES, VS2042) at 3000 g for 3 minutes. A non-stained control was treated equally. Phage titers of unstained and stained phages were then determined via double-layer plaque assay.

1.6 Resistant development to phages

Wells with bacterial regrowth were identified and content was transferred into a new tube. After a centrifugation step (5000 g for 3 minutes), supernatant was discarded and bacterial pellet was resuspended with LB media. Suspension was then streaked out on a plate and incubated

overnight at 37 °C. 17 individual colonies were picked from various plates and used to prepare overnight cultures. On the next day, 200 µL culture were mixed with LB soft agar (0.6 %) and poured onto a plate. 10 µL of serial diluted phage were spotted onto it and incubated at 37 °C overnight.

1.7 One-step-growth curve

Bacteria and phages were mixed at an MOI of 0.01 and 200 µL of that mixture were added into a 96-well plate. Plate was incubated at 37 °C and continuous shaking for an hour. Every five minutes 100 µL were taken out, mixed with 900 µL LB and filtered through a 0.22 µm syringe-filter. Samples were serially diluted, and phage titers were determined via plaque assay. Experiment was done in triplicates.

Supplementary Tables:

Table 1. Primers for multiplex qPCR

Primer_name	Sequence	Length	GC content	T_m	Product length
**E.coli_head_fow**	TAACGGTAACTCCGCAGAATG	21	47.6	62	116
**E.coli_head_rev**	GATCGCGTAGTCGCTGTAAATA	22	45.5	62	
**E.coli_eaeA_fow**	GCTAAAGCGGATGGCATAGA	20	50	62	107
**E.coli_eaeA_rev**	GCAGTCCCGGATACAATACTAAA	23	43.5	62	
**E.coli_toxin_fow_set2**	GGCCCGCATCCAGTTATG	18	61.1	63	85
**E.coli_toxin_rev_set2**	GCGAGTGACGGCTTTGT	17	58.8	62	
**E.coli_T4_head_fow**	GGTACGTCGTGCTATTCCTAAC	22	50	62	117
**E.coli_T4_head_rev**	GCCACTGGGTCTTTACCATATAC	23	47.8	62	
**E.coli_T4_eae_fow**	CTCAGGCTAATGTCCCTGTAAC	22	50	62	89
**E.coli_T4_eae_rev**	CTTACCGTTACCATCCGTTCTG	22	50	62	
**E.coli_T4_toxin_fow**	TCAACACAGTATATCCGAAGGC	22	45.5	62	100
**E.coli_T4_toxin_rev**	GTGACGGCTTTGTAGTCCTT	20	50	62	
**E.coli_T7_head_fow**	CACGTCTTCCCTGCCAATAA	20	50	62	104
**E.coli_T7_head_rev**	CGCAGCTTAACAGTACCTACC	21	52.4	62	
**E.coli_T7_eaeA_fow**	GCTAAAGCGGATGGCATAGA	20	50	62	107
**E.coli_T7_eaeA_rev**	GCAGTCCCGGATACAATACTAAA	23	43.5	62	
**E.coli_T7_toxin_fow**	GGCCCGCATCCAGTTATG	18	61.1	63	85
**E.coli_T7_toxin_rev**	GCGAGTGACGGCTTTGT	17	58.8	62	
**E.coli_T1_head_fow**	TCGACGCGGTACAAACTAATATC	23	43.5	62	101

**E.coli_T1_head_rev**	GAACTCCTGTTCGGCATCAA	20	50	62	
**E.coli_T4_head_fow**	GGTACGTCGTGCTATTCCTAAC	22	50	62	117
**E.coli_T4_head_rev**	GCCACTGGGTCTTTACCATATAC	23	47.8	62	
**E.coli_T7_head_fow**	CACGTCTTCCCTGCCAATAA	20	50	62	104
**E.coli_T7_head_rev**	CGCAGCTTAACAGTACCTACC	21	52.4	62	

Table 2. Probes for multiplex qPCR

Probe_name	Sequence	Fluorophore / Quencher	Length	GC content	T_m
**E.coli_head_probe**	AAGGACGTTGTTGTCGGATCGTGT	Cy5 - BHQ3	24	50	68
**E.coli_eaeA_probe**	AATGGTGTAGCTCAGGCTAATGTCCC	HEX -BHQ1	26	50	68
**E.coli_toxin_probe_set2**	CATCGTGCATATGGTGCGCAACAG	FAM - BHQ1	24	54	68
**E.coli_T4_head_probe**	CGGCTGAACACCACAAATATCGAAAGC	Cy5 - BHQ3	27	48	68
**E.coli_T4_eae_probe**	CCCAAGAGTTGCAGTCCCGGATAC	HEX -BHQ1	24	58	68
**E.coli_T4_toxin_probe**	CATCGTGCATATGGTGCGCAACAG	FAM - BHQ1	24	54	68
**E.coli_T7_head_probe**	AGGTGAGGGTAATGTCAAGGTTGCT	Cy5 - BHQ3	25	48	68
**E.coli_T7_eaeA_probe**	AATGGTGTAGCTCAGGCTAATGTCCC	HEX -BHQ1	26	50	68
**E.coli_T7_toxin_probe**	CATCGTGCATATGGTGCGCAACAG	FAM - BHQ1	24	54	68
**E.coli_T1_head_probe**	AAAGGTCGTGCGGGAATTGCTAAA	Cy5 - BHQ3	24	46	67
**E.coli_T4_head_probe**	CGGCTGAACACCACAAATATCGAAAGC	HEX -BHQ1	27	48	68
**E.coli_T7_head_probe**	AGGTGAGGGTAATGTCAAGGTTGCT	FAM - BHQ1	25	48	68

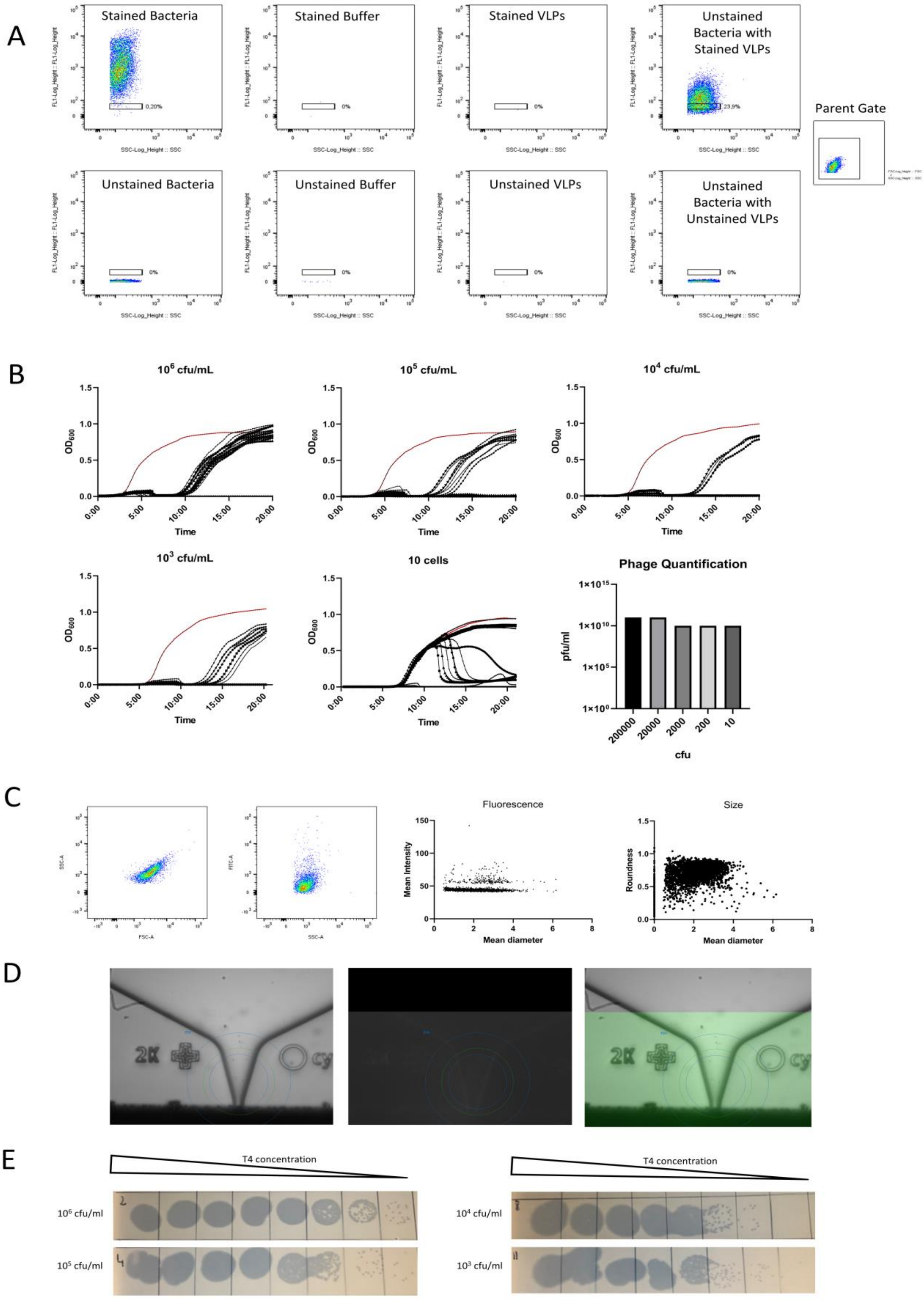
A
Stained Bacteria
0,20%
Stained Buffer
0%
Stained VLPs
0%
Unstained Bacteria with Stained VLPs
23,9%
Parent Gate
Unstained Bacteria
Unstained Buffer
Unstained VLPs
Unstained Bacteria with Unstained VLPs
FL1-Log_Height :: FL1-Log_Height
SSC-Log_Height :: SSC
B
10^6 cfu/mL
10^5 cfu/mL
10^4 cfu/mL
10^3 cfu/mL
10 cells
OD600
Time
Phage Quantification
pfu/ml
cfu
200000
20000
2000
200
10
C
SSC-A
FSC-A
FITC-A
Fluorescence
Mean Intensity
Mean diameter
Size
Roundness
D
E
T4 concentration
10^6 cfu/ml
10^5 cfu/ml
10^4 cfu/ml
10^3 cfu/ml

Supplementary Figure 1. Development of single-cell viral tagging on a microfluidics device

(A) Flow Cytometry plots from a viral tagging experiment: the top row shows stained samples, whereas the bottom row shows unstained samples. Bacterial, buffer and VLP controls are included. Plots were generated based on the parent gate shown on the right. The cytometer plot shows the side scatter on the x-axis and the fluorescence on the y-axis.

(B) Growth kinetics of four different bacterial concentrations: Kinetics were monitored of four different bacterial concentrations (10^6 CFU/mL, 10^5 CFU/mL, 10^4 CFU/mL, 10^3 CFU/mL and 10 sorted bacterial cells) with one sorted viral tagged-pair. Bacterial controls are shown in red, wells including bacteria and one viral tagged cell are in black. X-axis shows the time and y-axis the OD_{600} value. The bar plot shows different phage titers for different initial bacterial concentrations.

(C) Comparison of viral tagged sample: Viral tagged sample is shown on a flow cytometry plot (left side) and on a microfluidics device (right side) comparing once size vs surface appearance and once fluorescence vs size.

(D) bf.sight camera pictures: Microfluidics device analyses cells with two cameras and takes pictures in the brightfield mode (left), fluorescence mode(middle) and an overlay mode (right).

(E) Phage titer calculation: Spot assays were used to calculate phage titers which were between 10^9 PFU/mL and 10^{10} PFU/mL.

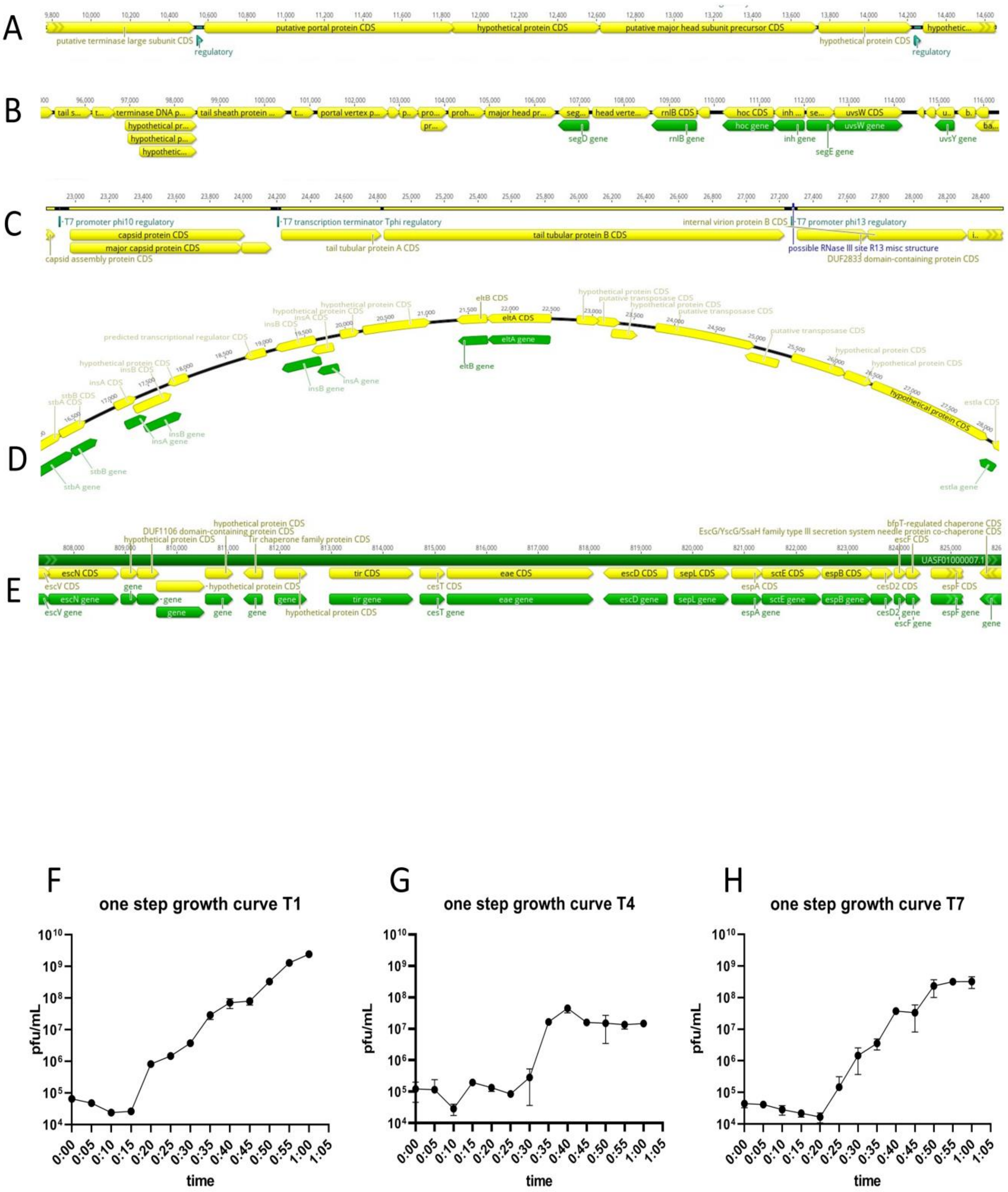
A
putative terminase large subunit CDS
regulatory
putative portal protein CDS
hypothetical protein CDS
putative major head subunit precursor CDS
B
terminase DNA p...
tail sheath protein ...
portal vertex p...
major head pr...
head verte...
rnlB CDS
hoc CDS
uvsW CDS
segD gene
rnlB gene
hoc gene
inh gene
uvsW gene
segE gene
uvsY gene
C
T7 promoter phi10 regulatory
T7 transcription terminator Tphi regulatory
internal virion protein B CDS
T7 promoter phi13 regulatory
capsid protein CDS
major capsid protein CDS
tail tubular protein B CDS
tail tubular protein A CDS
possible RNase III site R13 misc structure
capsid assembly protein CDS
DUF2833 domain-containing protein CDS
D
eltB CDS
eltA CDS
eltA gene
eltB gene
predicted transcriptional regulator CDS
putative transposase CDS
hypothetical protein CDS
insA CDS
insB CDS
stbA CDS
stbB CDS
insA gene
insB gene
stbA gene
stbB gene
estIa CDS
estIa gene
E
DUF1106 domain-containing protein CDS
Tir chaperone family protein CDS
EscG/YscG/SsaH family type III secretion system needle protein co-chaperone CDS
bfpT-regulated chaperone CDS
escN CDS
tir CDS
eae CDS
escD CDS
sepL CDS
sctE CDS
espB CDS
escV CDS
cesT CDS
espA CDS
cesD2 CDS
escF CDS
espF CDS
escV gene
escN gene
tir gene
eae gene
escD gene
sepL gene
sctE gene
espB gene
cesT gene
espA gene
cesD2 gene
escF gene
espF gene
F
one step growth curve T1
G
one step growth curve T4
H
one step growth curve T7
pfu/mL
time

Supplementary Figure 2. Genes for Primer Design

(A-C) Targeted phage genes: Primers were designed for T1 (A), T4 (B) and T7 (C) based on their structural head genes.

(D-E) Unwanted genes: toxin primer is based on toxin gene LT subunit A (D) and the virulence factor is based on eae gene (E).

(F-H) one-step-growth curves: each plot shows the time on the x-axis is minutes until one hours. The y-axis is pfu/mL. One-step-growth curve of T1 is shown in graph (F) with a latency period of 20minutes and a burst size of 19 on average. T4 is shown in graph (G) with a latency period of 35 minutes and a burst size of and 174 on average. In graph (H), T7 infected cells are shown which have a latency period of 30 minutes and a burst size of 22 on average.

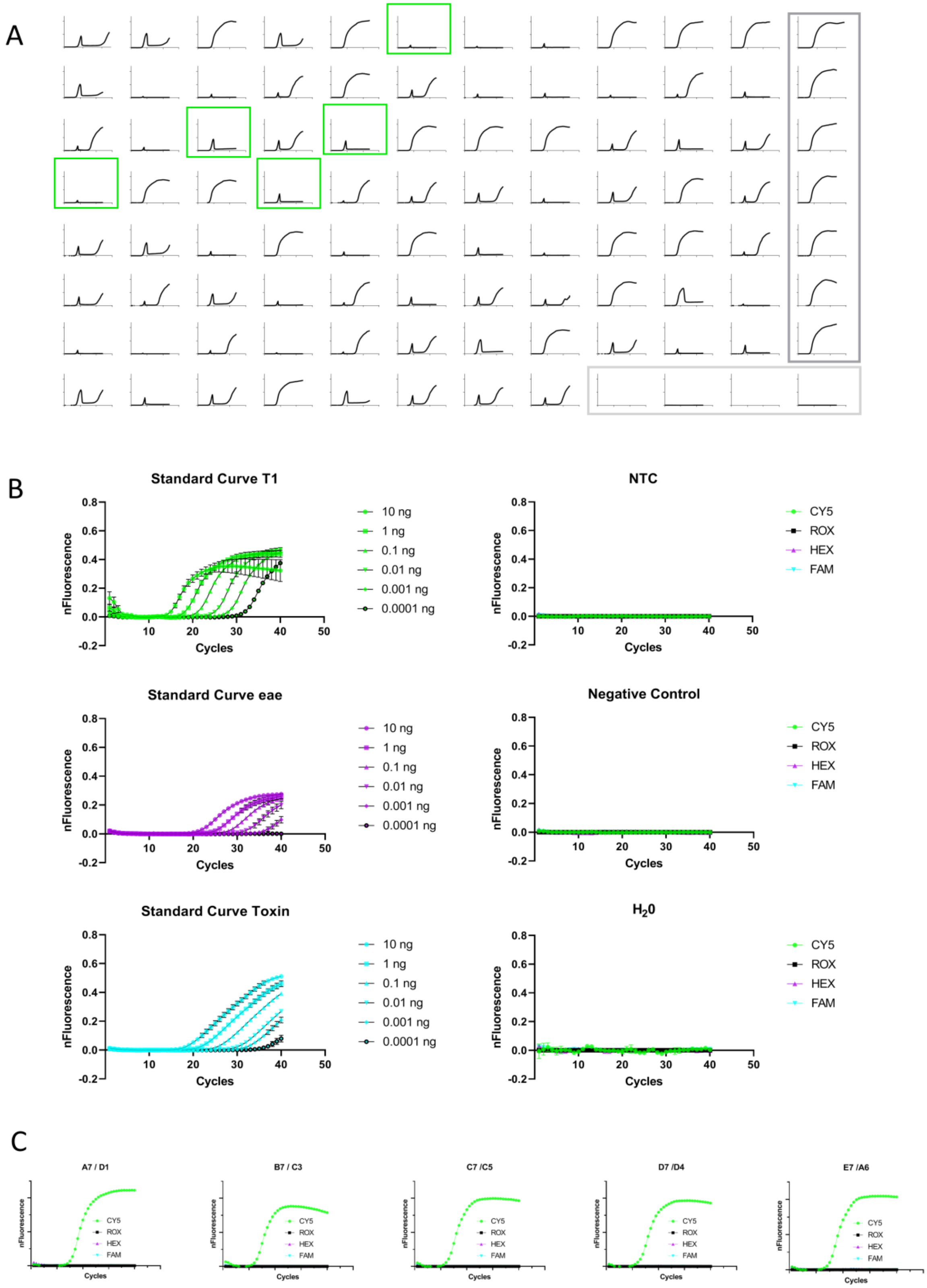
A
B
Standard Curve T1
nFluorescence
Cycles
10 ng
1 ng
0.1 ng
0.01 ng
0.001 ng
0.0001 ng
NTC
CY5
ROX
HEX
FAM
Standard Curve eae
Negative Control
Standard Curve Toxin
H$_2$0
C
A7 / D1
B7 / C3
C7 /C5
D7 /D4
E7 /A6

Supplementary Figure 3. Targeted Single Phage Isolation on T1

(A) Growth Kinetics: Kinetics monitoring for all 96-wells over 24 hours. Wells include 10 bacterial cell and one viral tagged pair. Cells were infected with T1. Bacterial controls are framed in dark grey, media controls in light grey. Green boxes are those samples which are shown in the qPCR plots in (C).

(B) Multiplex qPCR Controls: After kinetics monitoring samples were analyzed via multiplex qPCR. All standards were run in triplicates. T1 signal is shown in green, toxin gene amplification in turquoise and in pink the virulence factor eae. Each well included all primers and probes. Negative control included extracted lambda DNA 1 ng. No template control was performed with water.

(C) Targeted multiplex qPCR: Sample qPCR plots ran with qPCR mastermix including all primers and probes. Only the target is amplified and no unwanted or unspecific amplification happened. Each qPCR plot is connected with one green box in the 96-well kinetics monitoring at the top.

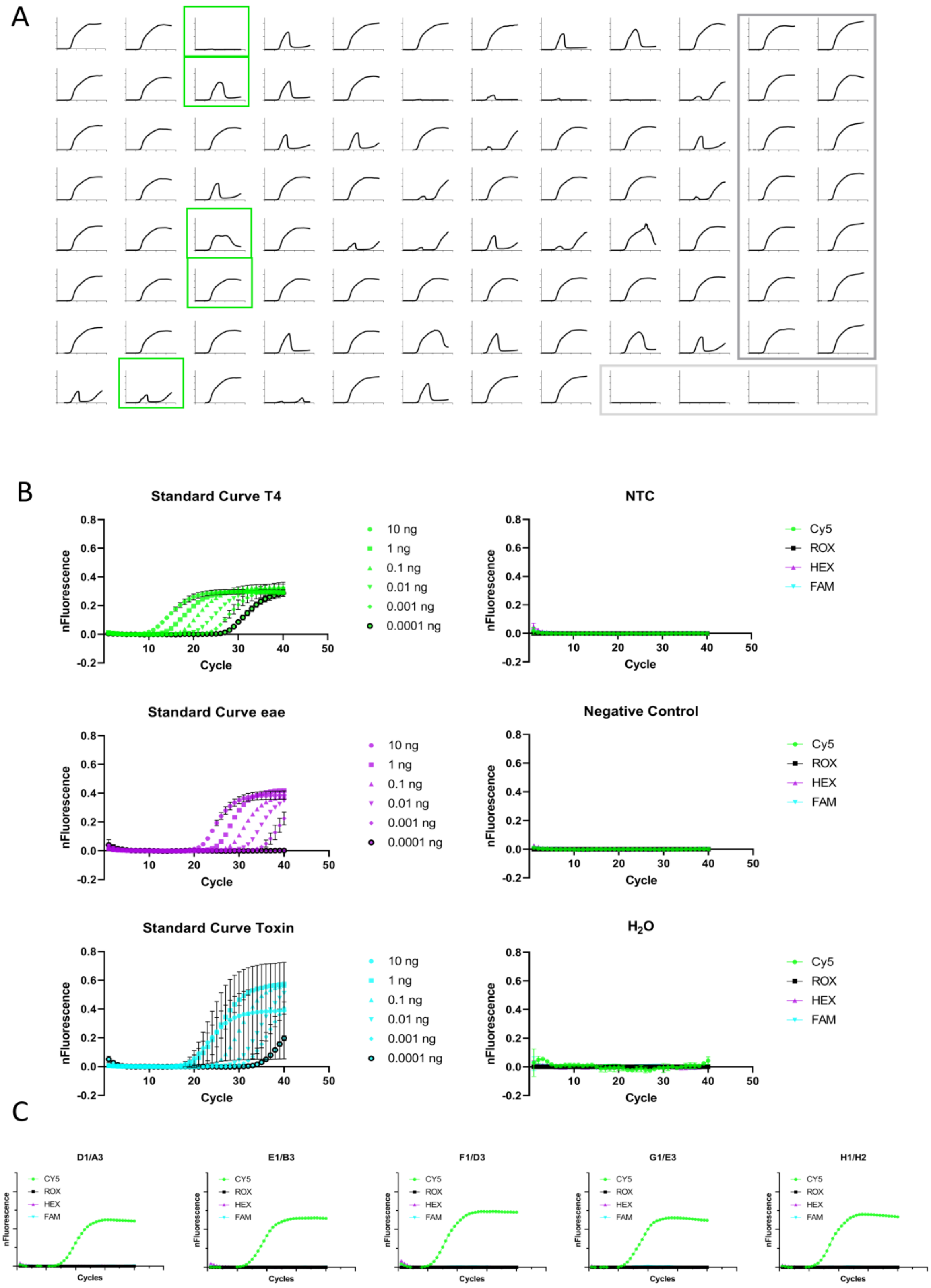
A
B
Standard Curve T4
10 ng
1 ng
0.1 ng
0.01 ng
0.001 ng
0.0001 ng
nFluorescence
Cycle
NTC
Cy5
ROX
HEX
FAM
Standard Curve eae
Negative Control
Standard Curve Toxin
H2O
C
D1/A3
E1/B3
F1/D3
G1/E3
H1/H2
CY5
ROX
HEX
FAM
nFluorescence
Cycles

Supplementary Figure 4. Targeted Single Phage Isolation on T4

(A) Growth Kinetics: Kinetics monitoring for all 96-wells over 24 hours. Wells include 10 bacterial cell and one viral tagged pair. Cells were infected with T4. Bacterial controls are framed in dark grey, media controls in light grey. Green boxes are those samples which are shown in the qPCR plots in (C).

(B) Multiplex qPCR Controls: After kinetics monitoring samples were analyzed via multiplex qPCR. All standards were run in triplicates. T4 signal is shown in green, toxin gene amplification in turquoise and in pink the virulence factor eae. Each well included all primers and probes. Negative control included extracted lambda DNA 1 ng. No template control was performed with water.

(C) Targeted multiplex qPCR: Sample qPCR plots ran with qPCR mastermix including all primers and probes. Only the target is amplified and no unwanted or unspecific amplification happened. Each qPCR plot is connected with one green box in the 96-well kinetics monitoring at the top.

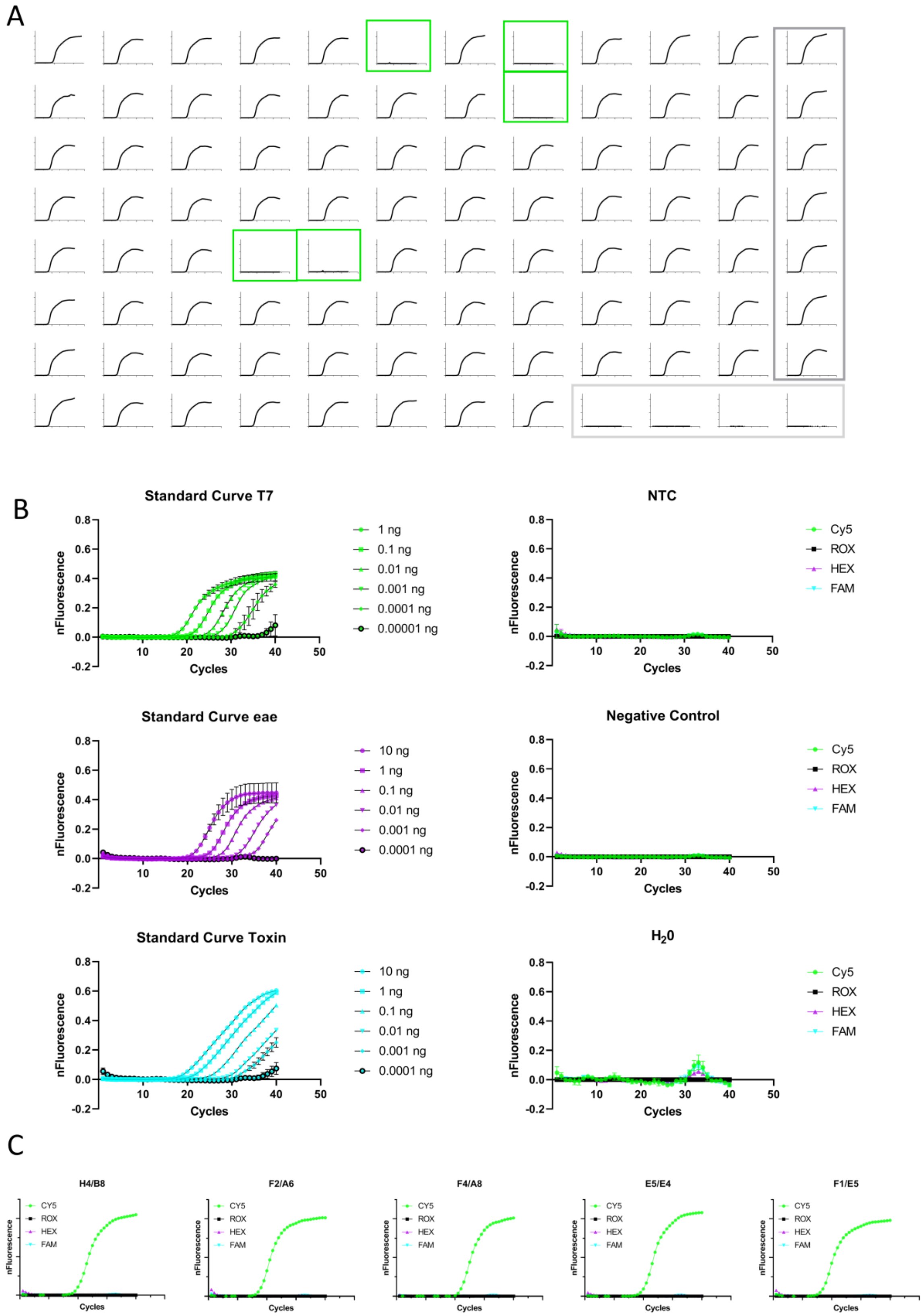
A
B
Standard Curve T7
1 ng
0.1 ng
0.01 ng
0.001 ng
0.0001 ng
0.00001 ng
NTC
Cy5
ROX
HEX
FAM
Standard Curve eae
10 ng
1 ng
0.1 ng
0.01 ng
0.001 ng
0.0001 ng
Negative Control
Standard Curve Toxin
H20
nFluorescence
Cycles
C
H4/B8
F2/A6
F4/A8
E5/E4
F1/E5
CY5

Supplementary Figure 5. Targeted Single Phage Isolation on T7

(A) Growth Kinetics: Kinetics monitoring for all 96-wells over 24 hours. Wells include 10 bacterial cell and one viral tagged pair. Cells were infected with T7. Bacterial controls are framed in dark grey, media controls in light grey. Green boxes are those samples which are shown in the qPCR plots in (C).

(B) Multiplex qPCR Controls: After kinetics monitoring samples were analyzed via multiplex qPCR. All standards were run in triplicates. T7 signal is shown in green, toxin gene amplification in turquoise and in pink the virulence factor eae. Each well included all primers and probes. Negative control included extracted lambda DNA 1 ng. No template control was performed with water.

(C) Targeted multiplex qPCR: Sample qPCR plots ran with qPCR mastermix including all primers and probes. Only the target is amplified and no unwanted or unspecific amplification happened. Each qPCR plot is connected with one green box in the 96-well kinetics monitoring at the top.

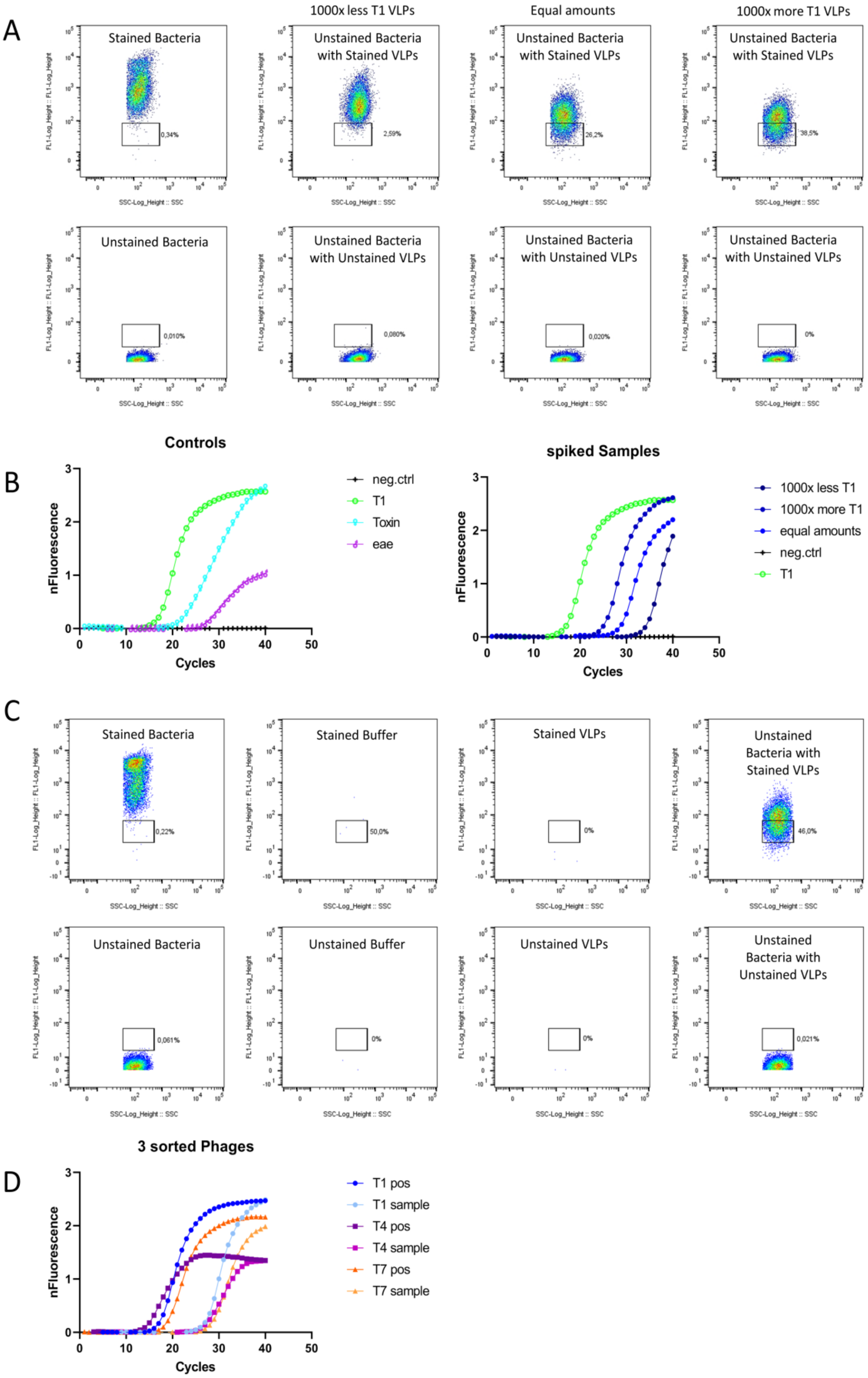
A
1000x less T1 VLPs
Equal amounts
1000x more T1 VLPs
Stained Bacteria
Unstained Bacteria with Stained VLPs
Unstained Bacteria
Unstained Bacteria with Unstained VLPs
0,34%
2,59%
26,2%
38,5%
0,010%
0,080%
0,020%
0%
FL1-Log_Height :: FL1-Log_Height
SSC-Log_Height :: SSC
B
Controls
spiked Samples
nFluorescence
Cycles
neg.ctrl
T1
Toxin
eae
1000x less T1
1000x more T1
equal amounts
neg.ctrl
T1
C
Stained Bacteria
Stained Buffer
Stained VLPs
Unstained Bacteria with Stained VLPs
Unstained Bacteria
Unstained Buffer
Unstained VLPs
Unstained Bacteria with Unstained VLPs
0,22%
50,0%
0%
46,0%
0,061%
0%
0%
0,021%
D
3 sorted Phages
nFluorescence
Cycles
T1 pos
T1 sample
T4 pos
T4 sample
T7 pos
T7 sample

Supplementary Figure 6. qPCR Specificity Testing

(A) Spiked wastewater sorting: Samples on Flow Cytometer: Escherichia coli cells mixed with spiked wastewater. First column shows stained and unstained E. coli (far left). Second column shows wastewater spiked with 1000 T1 VLPs less than wastewater VLPs. Third column has equal amounts of wastewater VLPs and T1 included. The last column (far right) has 1000x more T1 VLPs than wastewater VLPs included.

(B) Spiked wastewater qPCR: Bulk sorted cells were analyzed via multiplex qPCR. On the left, all qPCR controls - T1 in green, toxin in turquoise and virulence factor in purple. On the right areT1 DNA as a positive control in green, and the three different concentrations of spiked wastewater in blue shades. In black is the negative control.

(C)3 sorted phages Flow Cytometry: E. coli cells were combined with a mixture of T1, T4 and T7 in equal concentrations. Far left shows stained vs unstained bacteria. Buffer and VLPs controls are shown in the middle. Far right shows viral tagged sample (top) and E. coli with unstained VLPs (bottom).

(D)3 sorted phages qPCR. Flow cytometry sorted phages were identified via qPCR with their distinct primers. The qPCR plot shows on the y-axis the fluorescence level and on the x-axis the amount of cycles. For each phage a DNA extracted control was run as well. T1 sample is shown in light blue and its control in dark blue. T4 control is in dark purple and its sample in light purple. T7 control is presented in dark orange and its sample in light orange.

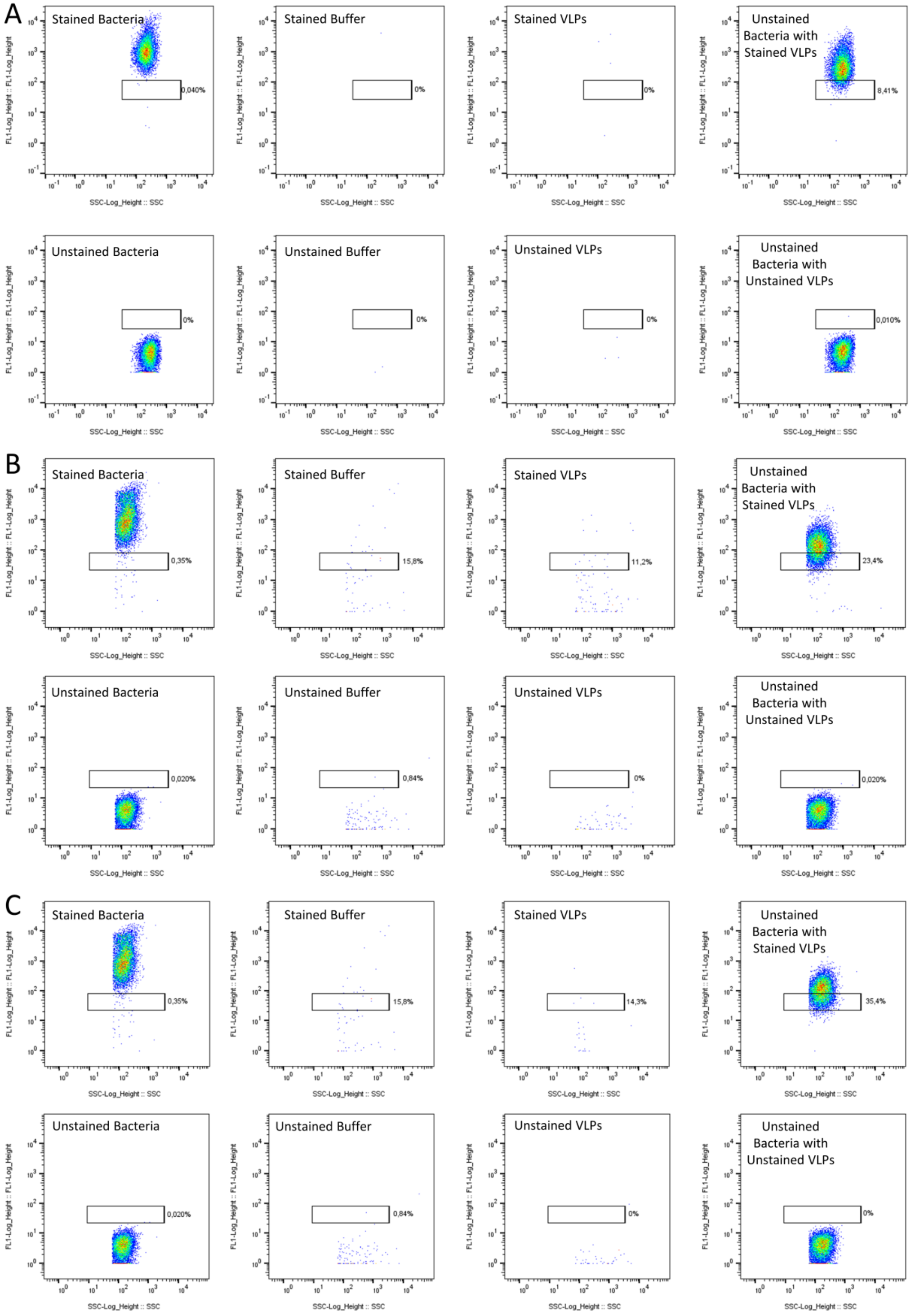
A
Stained Bacteria
0,040%
Stained Buffer
0%
Stained VLPs
0%
Unstained Bacteria with Stained VLPs
8,41%
Unstained Bacteria
0%
Unstained Buffer
0%
Unstained VLPs
0%
Unstained Bacteria with Unstained VLPs
0,010%
B
Stained Bacteria
0,35%
Stained Buffer
15,8%
Stained VLPs
11,2%
Unstained Bacteria with Stained VLPs
23,4%
Unstained Bacteria
0,020%
Unstained Buffer
0,84%
Unstained VLPs
0%
Unstained Bacteria with Unstained VLPs
0,020%
C
Stained Bacteria
0,35%
Stained Buffer
15,8%
Stained VLPs
14,3%
Unstained Bacteria with Stained VLPs
35,4%
Unstained Bacteria
0,020%
Unstained Buffer
0,84%
Unstained VLPs
0%
Unstained Bacteria with Unstained VLPs
0%
FL1-Log_Height :: FL1-Log_Height
SSC-Log_Height :: SSC

Supplementary Figure 7 Flow Cytometry Plots of Spiked Wastewater

(A) 1000x less T1 VLPs than wastewater VLPs: Escherichia coli cells mixed with wastewater which was spiked with 1000x less T1 VLPs than wastewater VLPs. First column shows stained and unstained E. coli (far left). Buffer and VLPs controls are shown in the middle. Far right shows viral tagged sample (top) and E. coli with unstained VLPs (bottom).

(B) Equal amounts of VLPs: Escherichia coli cells mixed with wastewater which was spiked with equal amounts of VLPs. First column shows stained and unstained E. coli (far left). Buffer and VLPs controls are shown in the middle. Far right shows viral tagged sample (top) and E. coli with unstained VLPs (bottom).

(C) 1000x more T1 VLPs than wastewater VLPs: Escherichia coli cells mixed with wastewater which was spiked with 1000x more T1 VLPs than wastewater VLPs. First column shows stained and unstained E. coli (far left). Buffer and VLPs controls are shown in the middle. Far right shows viral tagged sample (top) and E. coli with unstained VLPs (bottom).

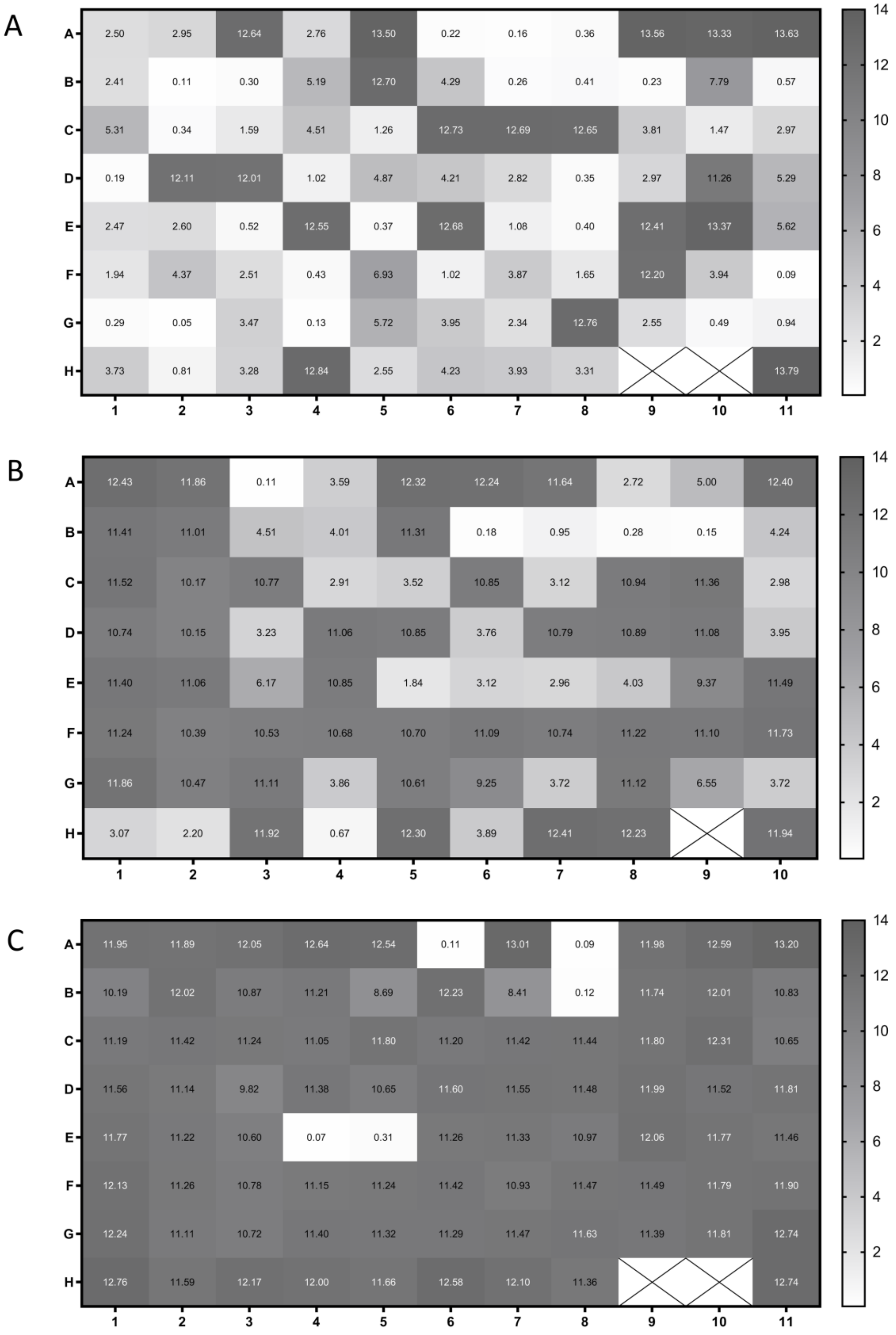
A
A 2.50 2.95 12.64 2.76 13.50 0.22 0.16 0.36 13.56 13.33 13.63
B 2.41 0.11 0.30 5.19 12.70 4.29 0.26 0.41 0.23 7.79 0.57
C 5.31 0.34 1.59 4.51 1.26 12.73 12.69 12.65 3.81 1.47 2.97
D 0.19 12.11 12.01 1.02 4.87 4.21 2.82 0.35 2.97 11.26 5.29
E 2.47 2.60 0.52 12.55 0.37 12.68 1.08 0.40 12.41 13.37 5.62
F 1.94 4.37 2.51 0.43 6.93 1.02 3.87 1.65 12.20 3.94 0.09
G 0.29 0.05 3.47 0.13 5.72 3.95 2.34 12.76 2.55 0.49 0.94
H 3.73 0.81 3.28 12.84 2.55 4.23 3.93 3.31 13.79
1 2 3 4 5 6 7 8 9 10 11
14 12 10 8 6 4 2
B
A 12.43 11.86 0.11 3.59 12.32 12.24 11.64 2.72 5.00 12.40
B 11.41 11.01 4.51 4.01 11.31 0.18 0.95 0.28 0.15 4.24
C 11.52 10.17 10.77 2.91 3.52 10.85 3.12 10.94 11.36 2.98
D 10.74 10.15 3.23 11.06 10.85 3.76 10.79 10.89 11.08 3.95
E 11.40 11.06 6.17 10.85 1.84 3.12 2.96 4.03 9.37 11.49
F 11.24 10.39 10.53 10.68 10.70 11.09 10.74 11.22 11.10 11.73
G 11.86 10.47 11.11 3.86 10.61 9.25 3.72 11.12 6.55 3.72
H 3.07 2.20 11.92 0.67 12.30 3.89 12.41 12.23 11.94
1 2 3 4 5 6 7 8 9 10
14 12 10 8 6 4 2
C
A 11.95 11.89 12.05 12.64 12.54 0.11 13.01 0.09 11.98 12.59 13.20
B 10.19 12.02 10.87 11.21 8.69 12.23 8.41 0.12 11.74 12.01 10.83
C 11.19 11.42 11.24 11.05 11.80 11.20 11.42 11.44 11.80 12.31 10.65
D 11.56 11.14 9.82 11.38 10.65 11.60 11.55 11.48 11.99 11.52 11.81
E 11.77 11.22 10.60 0.07 0.31 11.26 11.33 10.97 12.06 11.77 11.46
F 12.13 11.26 10.78 11.15 11.24 11.42 10.93 11.47 11.49 11.79 11.90
G 12.24 11.11 10.72 11.40 11.32 11.29 11.47 11.63 11.39 11.81 12.74
H 12.76 11.59 12.17 12.00 11.66 12.58 12.10 11.36 12.74
1 2 3 4 5 6 7 8 9 10 11
14 12 10 8 6 4 2

Supplementary Figure 8 Phage-Bacteria Infection Dynamics

(A-C) Infection dynamics visualized as a heatmap: AUC values are presented as a heatmap mocking a 96-well plate. Cells infected with T1 are shown in A, with T4 in B and T7 in C. The color scale shows the AUC measurement for each well, ranging from 0 to 14. Bacterial controls are marked with a C. Wells with an X are empty.

(D-F) one-step-growth curves: each plot shows the time on the x-axis is minutes until one hours. The y-axis are pfu/mL. One-step-growth curve of T1 is shown in graph (D), of T4 in graph (E) and for T7 in graph (F).

Supplementary References:

1. Boling L, Cuevas DA, Grasis JA, Kang HS, Knowles B, Levi K, Maughan H, McNair K, Rojas MI, Sanchez SE, Smurthwaite C, Rohwer F. Dietary prophage inducers and antimicrobials: toward landscaping the human gut microbiome. Gut Microbes 11:721–734.
